# Exercise Increases Markers of Spermatogenesis in Rats Selectively Bred for Low Running Capacity

**DOI:** 10.1371/journal.pone.0114075

**Published:** 2014-12-10

**Authors:** Ferenc Torma, Erika Koltai, Enikő Nagy, Mohammad Mosaferi Ziaaldini, Aniko Posa, Lauren G. Koch, Steven L. Britton, Istvan Boldogh, Zsolt Radak

**Affiliations:** 1 Research Institute of Sport Science, Semmelweis University, Budapest, Hungary; 2 Department of Physiology, Anatomy and Neuroscience, Faculty of Science and Informatics, University of Szeged, Szeged, Hungary; 3 Department of Anesthesiology, University of Michigan Medical School, Ann Arbor, Michigan, United States of America; 4 Department of Microbiology and Immunology, Sealy Center for Molecular Medicine, University of Texas Medical Branch at Galveston, Galveston, Texas, United States of America; Pennington Biomedical Research Center, United States of America

## Abstract

The oxidative stress effect of exercise training on testis function is under debate. In the present study we used a unique rat model system developed by artificial selection for low and high intrinsic running capacity (LCR and HCR, respectively) to evaluate the effects of exercise training on apoptosis and spermatogenesis in testis. Twenty-four 13-month-old male rats were assigned to four groups: control LCR (LCR-C), trained LCR (LCR-T), control HCR (HCR-C), and trained HCR (HCR-T). Ten key proteins connecting aerobic exercise capacity and general testes function were assessed, including those that are vital for mitochondrial biogenesis. The VO2 max of LCR-C group was about 30% lower than that of HCR-C rats, and the SIRT1 levels were also significantly lower than HCR-C. Twelve weeks of training significantly increased maximal oxygen consumption in LCR by nearly 40% whereas HCR remained unchanged. LCR-T had significantly higher levels of peroxisome proliferator-activated receptor-gamma coactivator-1 (PGC-1α), decreased levels of reactive oxygen species and increased acetylated p53 compared to LCR-C, while training produced no significant changes for these measures in HCR rats. BAX and Blc-2 were not different among all four groups. The levels of outer dense fibers -1 (Odf-1), a marker of spermatogenesis, increased in LCR-T rats, but decreased in HCR-TR rats. Moreover, exercise training increased the levels of lactate dehydrogenase C (LDHC) only in LCR rats. These data suggest that rats with low inborn exercise capacity can increase whole body oxygen consumption and running exercise capacity with endurance training and, in turn, increase spermatogenesis function via reduction in ROS and heightened activity of p53 in testes.

## Introduction

There is accumulating evidence that a sedentary lifestyle can adversely affect spermatogenesis in the testis [1], [2]. However, data on the effects of exercise on testosterone and spermatogenesis are diverse. For example, reports show long term endurance exercise training can decrease the production of testosterone [3], which in turn, could associate with a decrease in spermatogenesis [4], [5]. Indeed, the testosterone levels of endurance trained athletes can be 30–40% lower than those of age matched controls [6]. Studies using animal models show that swimming 3 h per day, which is considered intensive exercise, results in testicular gametogenic and steroidogenic disorders and increased oxidative stress in rats [7]. On the other hand, lifelong voluntary wheel running decreased the accumulation of oxidative damage and attenuated the age-associated decrease in spermatogenesis in testes of mice [4].

Seven mammalian homologs of silent information regulator (SIRTs 1–7) proteins belong to a family of evolutionarily conserved NAD^+^-dependent enzymes with deacetylase and/or mono-ADP ribosyltransferase activity [8]. Sirtuins are implicated in diverse cellular processes including the regulation of apoptosis by the deacetylation of p53 [9] followed by down-stream regulation of Bax [10]. Besides the role in apoptosis, SIRT1 has an anti-inflammatory role in testis, which could influence spermatogenesis [11]. It has been shown that the absence of SIRT1 significantly attenuates spermatogenesis in mice [12], which suggests that SIRT1-mediated pathways are important to the physiology of testis. In previous work we showed that physical exercise readily alters the activity of SIRT1 and the content of SIRT3, SIRT4 and SIRT6 in various tissues [8]. Hence, it cannot be ruled out that the sirtuin level, which is altered by regular exercise in testis, could impact spermatogenesis.

Aerobic capacity can be divided into two components: an inborn aerobic capacity that occurs in the non-trained or sedentary condition and an adaptational capacity acquired in response to exercise training, An experimental animal model was created, based on the intrinsic (i.e. not trained) running capacity for rats [13]. Starting with the genetically heterogeneous rat population (N/NIH) as founders, rat lines of low capacity runners (LCR) and high capacity runners (HCR) were developed by artificial selective breeding. Besides the expected wide differential for running capacity (8-fold), the LCR rats have shorter life-spans, demonstrate increased risk for metabolic syndrome, obesity, and oxidative stress. [14], [15], [16] This model permits the study of the effects of different inherent levels of aerobic capacity and the effects of physical training on the levels of oxidative stress, apoptosis, and markers of spermatogenesis in the testis. It was hypothesized that high intrinsic running capacity and increased exercise capacity from physical training would beneficially affect apoptosis and spermatogenesis.

## Materials and Methods

### Animals

Selectively bred rat strains differing in intrinsic aerobic capacity – low capacity runners (LCR) and high capacity runners (HCR) – were used in this study [13]. Endurance running capacity was assessed on a treadmill and the total distance run during a speed-ramped exercise test was used as a measure of maximal aerobic capacity. Rats with the highest running capacity from each generation were bred to produce the HCR strain and rats with poor running capacity were bred to generate the LCR strain. A subgroup of male rats from generation 22 were phenotyped for intrinsic treadmill running capacity at 11 weeks of age, at the University of Michigan (Ann Arbor, MI, USA), and then shipped via air freight to Semmelweis University (Budapest, Hungary) for further study. Investigations were carried out according to the requirements of The Guiding Principles for Care and Use of Animals, EU, and approved by The Ethics Committee of Semmelweis University.

### Exercise protocol

Twenty-four LCR and HCR male, 13-month-old rats were assigned to groups as follows: control LCR (LCR-C), trained LCR (LCR-T), control HCR (HCR-C), and trained HCR (HCR-T). Exercised rats (six animals per group) were introduced to treadmill running for three days, and then for the next two weeks the running speed was set to 10 m/min on a 5% incline for 30 min. The maximal oxygen uptake (VO_2_max) was measured on the treadmill (Columbus Inst. Columbus, Ohio) with a gradually increasing intensity. VO_2_max was measured for each animal by using three criteria: (i) no change in VO_2_ when speed was increased, (ii) rats could no longer keep their position on the treadmill, and (iii) respiratory quotient (RQ = VCO_2_/VO_2_)>1. Then, based on the level of VO_2_max, the speed corresponding to 60% VO_2_max was determined and used for daily training for one hour, five times a week. The VO_2_max was measured every other week, and running speed was adjusted to maintain 60% VO_2_max [16]. The running distance was measured during each VO_2_max test. The training period lasted for 12 weeks. Animals were sacrificed and the testes were excised and frozen in liquid nitrogen until assayed for the measures described below.

### ROS measurements

Intracellular oxidant and redox-active iron levels [17] were estimated using modifications of the dichloro-dihydrofluorescein-diacetate (H_2_DCFDA) staining method [18]. The oxidative conversion of stable, non-fluorometric DCF-DA to highly fluorescent 2′7′-dichlorofluoorescein (DCF) was measured in the presence of esterases, as previously reported [18]. This assay approximates levels of reactive species, such as superoxide radical, hydroxyl radical, and hydrogen peroxide. The method has been widely used in the literature but does have the problem of not being particularly specific, and results can be strongly affected by the release of labile iron or copper [17]. Briefly, the H_2_DCFDA (Invitrogen-Molecular Probes #D399) was diluted to a concentration of 12.5 mM in ethanol and kept at −80°C in the dark. The solution was freshly diluted with potassium phosphate buffer to 125 µM before use. For fluorescence reactions, 96-well, black microplates were loaded with potassium phosphate buffer (pH 7.4) to a final concentration of 152 µM/well. Then 8 µl diluted tissue homogenate and 40 µl of 125 µM dye were added to achieve a final dye concentration of 25 µM. The change in fluorescence intensity was monitored every five minutes for 30 minutes with excitation and emission wavelengths set at 485 nm and 538 nm (Fluoroskan Ascent FL) respectively. Data obtained after 15 min were used for the analysis. The fluorescence intensity unit was normalized to the protein content and expressed in relative unit production per minute. The protein carbonyl measurement was done as described previously [19].

### Western blots

Ten to 50 micrograms of protein were electrophoresed on 8–12% v/v polyacrylamide SDS-PAGE gels. Proteins were electrotransfered onto PVDF membranes. The membranes were subsequently blocked and incubated at room temperature with antibodies (1∶500 #sc-13067 Santa Cruz PGC-1 (H-300), 1∶2000 #ab110304 Abcam Sirt-1, 1∶2000 #sc-99 Santa Cruz p53 (Pab 240), 1∶2000 #06–758 Upstate ac.p53 (Lys 373,382), 1∶1000 #sc-33771 Santa Cruz NRF-1 (H-300), 1∶500 #sc-30963 Santa Cruz mtTFA (E-16)/TFAM/, 1∶1000 #sc-492 Santa Cruz Blc-2 (N-19), 1∶1000 #sc-525 Santa Cruz Bax (P-19), 1∶1000 #sc-27907 Santa Cruz Odf-1 (G-17), 1∶2000 #sc-377305 Santa Cruz LDH-C (H-65), 1: 5000#sc1616 Santa Cruz Actin (I-19). After incubation with primary antibodies, membranes were washed in TBS-Tween-20 and incubated with HRP-conjugated secondary antibodies. After incubation with the secondary antibody, membranes were repeatedly washed. Membranes were incubated with an ECL Plus reagent (RPN 2132, Amersham) and protein bands were visualized on X-ray films. The bands were quantified by ImageJ software (http://imagej.nih.gov/ij/) and normalized to beta-actin, which served as an internal control.

### Statistical analyses

The results were compared with a Kruskal-Wallis analysis of variance (ANOVA) followed by Tukey’s post hoc test. Significance levels are reported for p<0.05.

## Results

**Figure 1:**
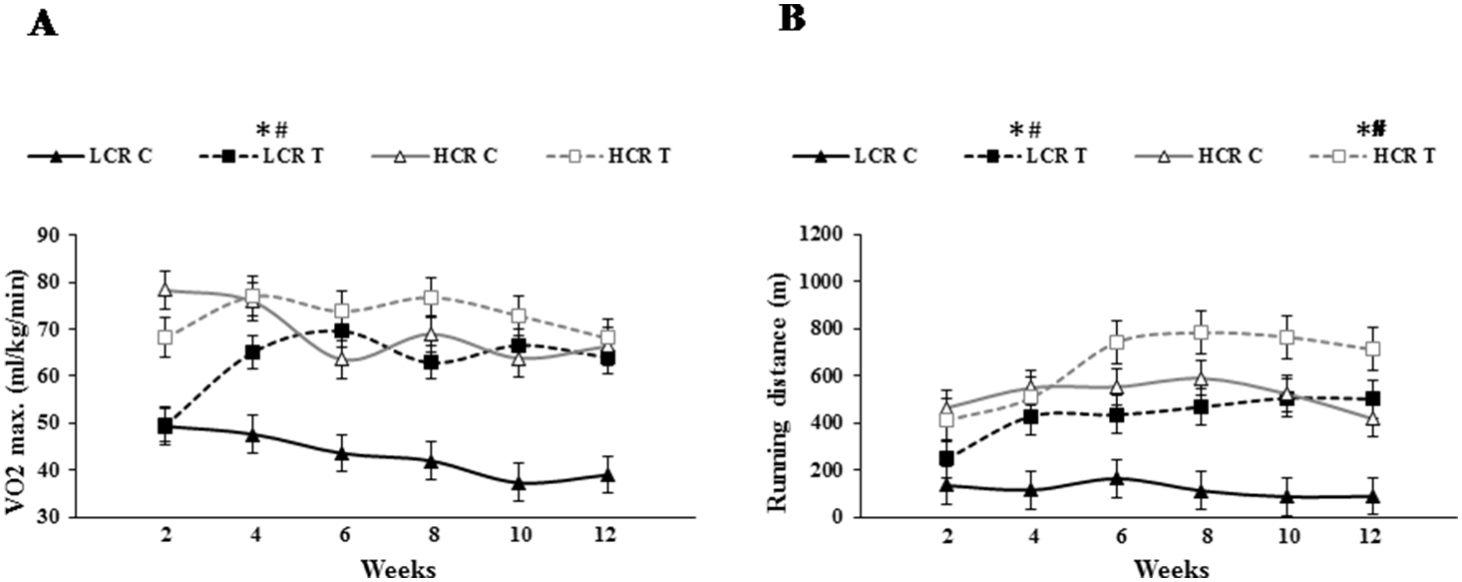
Running distance (A), VO2max (B) of LCR rats. Maximal relative oxygen uptake (VO_2_max; ml/kg/min)(**A**), and running distance (m) (**B**), for low capacity runner (LCR) and high capacity runner rats were measured every second week across a 12 week exercise training period and during the two weeks of treadmill habituation. Control LCR (LCR-C), trained LCR (LCR-T), high capacity control (HCR-C) and trained (HCR-T) groups. Values are means ± SD for six animals per group *Significantly different from control group, +Significantly different from trained group, #Significantly different fromthe starting value, p<0.05.

There was a significant difference in VO2max (Fig. 1A) and endurance running distance (Fig. 1B) between LCR and HCR rats at the beginning of the training program. The twelve-week running program significantly increased VO2max in the LCR strain to levels similar to HCR-C and HCR-T groups but VO2max did not increase in the HCR with training. Endurance running distance increased with training for both LCR and HCR rats suggesting that the trainability of LCR and HCR rats was similar.

**Figure 2:**
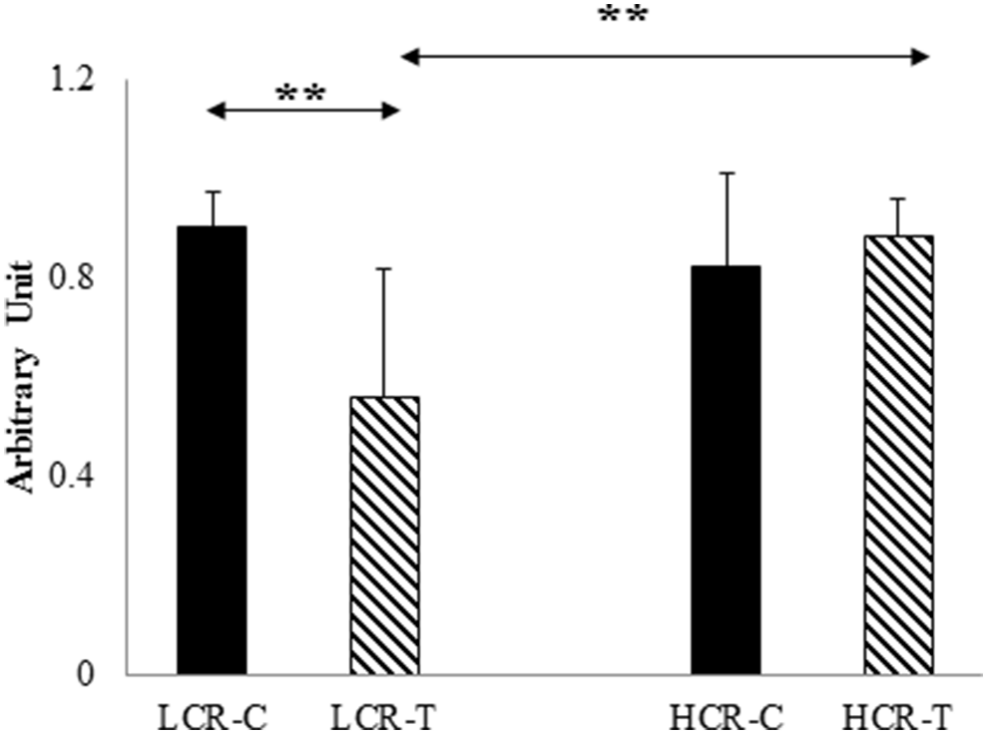
Oxidative stress markers. Rat gastrocnemius muscle was stained with dichlorodihydrofluoresceindiacetate (H_2_DCFDA) to measure relative steady-state oxidant levels and redox-active iron release levels (both increase DCF fluorescence) as an estimate of levels of reactive oxygen species (ROS).Control LCR (LCR-C), trained LCR (LCR-T), high capacity control (HCR-C) and trained (HCR-T) groups. Values are means ± SD for six animals per group *Significantly different from control group, +Significantly different from trained group, p<0.05.

**Figure 3:**
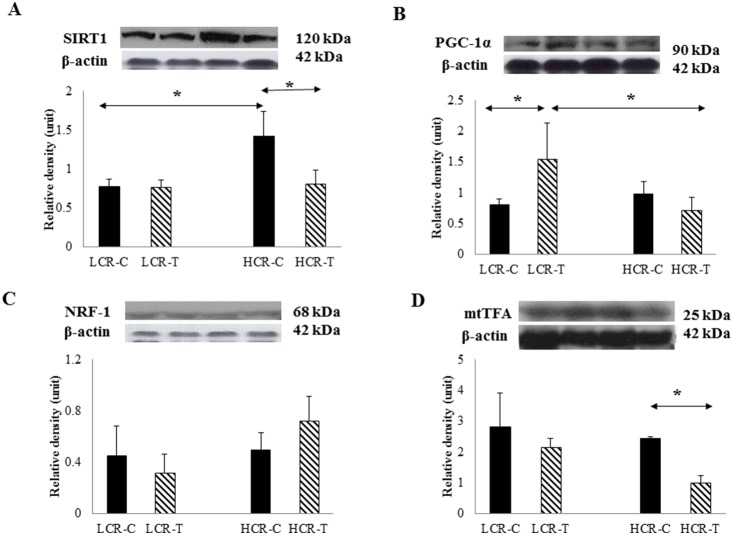
SIRT1 and mitochondrial biogenesis associated factors. SIRT1 levels of HCR-C rats were significantly elevated compared to LCR- C (**A**). On the other hand the PGC-1a levels were elevated in LCR-T rats compared to LCR-C or HCR-T (**B**). NRF1 concentration was unchanged (**C**), while mtTFA levels were attenuated by exercise in HCR-T group compared to HCR-C (**D**). Control LCR (LCR-C), trained LCR (LCR-T), high capacity control (HCR-C) and trained (HCR-T) groups. Values are means ± SD for six animals per group *Significantly different from control group, +Significantly different from trained group, p<0.05.

The level of testicular ROS was appraised by fluorescence intensity of H_2_DCFDA and exercise training decreased the level of ROS only in LCR-T rats compared to HCR-T group (Fig. 2). SIRT1 levels were significantly higher in HCR-C group compared to the LCR-C group and training decreased the protein content of HCR rats (Fig. 3A).Since SIRT1 can deacetylate PGC-1α, we checked the protein content of this transcriptional co-activator and found PGC-1α increased only in the LCR-T group (p<0.05, Fig. 3B). The levels of NRF1 were not different for any of the groups (Fig. 3C). On the other hand, testicular mtTFA levels were decreased only in the HCR-T group (Fig. 3D).

**Figure 4:**
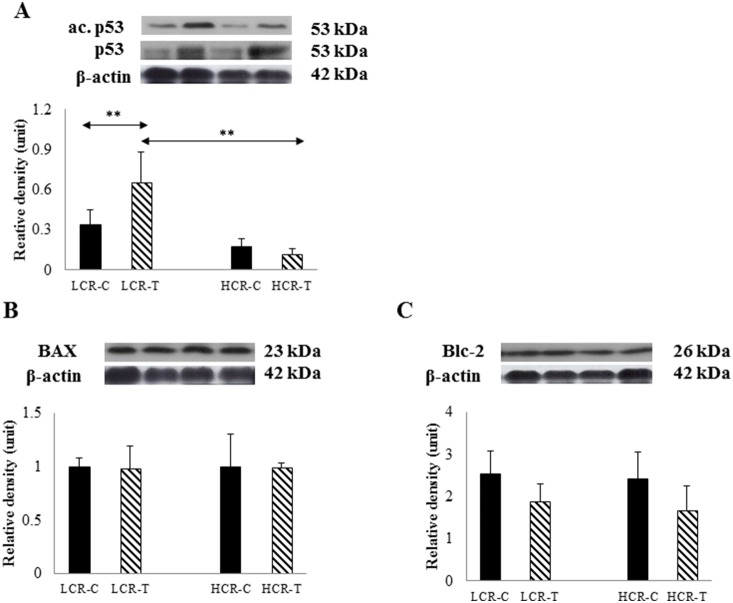
Apoptosis markers. The ratio of acetylated p53/and total p53 (**A**) levelsrelated to genetic stability.Bax (**B**) and Blc-2 (**C**) were evaluated to assess the levels of apoptosis. Control LCR (LCR-C), trained LCR (LCR-T), high capacity control (HCR-C) and trained (HCR-T) groups. Values are means ± SD for six animals per group *Significantly different from control group, +Significantly different from trained group, p<0.05.

**Figure 5:**
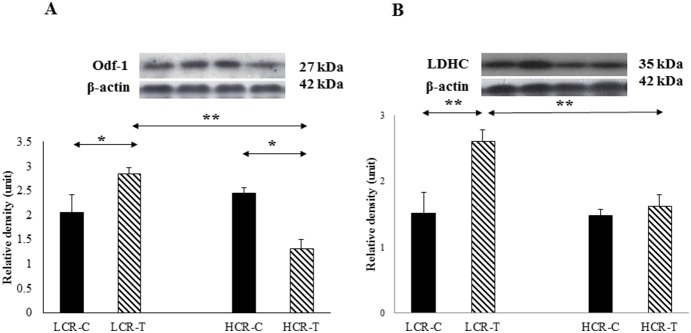
Markers of spermatogenesis. Outer dense fibers-1 (Odf-1) (**A**) and lactate dehydrogenase C (**B**) were detected by immunoblot assay. Control LCR (LCR-C), trained LCR (LCR-T), high capacity control (HCR-C) and trained (HCR-T) groups. Values are means ± SD for six animals per group *Significantly different from control group, +Significantly different from trained group, p<0.05.

Apoptosis factors were evaluated and the ratio of acp53/p53 was measured. p53 increased significantly with training in LCR rats, while a training effect was observed inHCR rats(Fig. 4A). Significant changes in the levels of Bax (Fig. 4B), and Bcl-2, were not detected. However, in the latter case, training tended to decrease the levels of Bcl-2 in both exercise trained groups (Fig. 4C). Odf1 proteins were synthesized and assembled in the cytoplasm of elongated spermatid and used as a marker of spermiogenesis [20]. The Odf1 levels increased significantly with training in the LCR group and decreased in the HCR rats (Fig. 5A). LDHC is a testis specific enzyme and exercise training increased the levels in LCR-T rats (Fig. 5B).

## Discussion

One of the important findings of this study is that the acetylation level of p53, a tissue specific tumor suppressor that mediates apoptosis, was significantly higher in rats with low intrinsic running capacity after training than rats with high intrinsic running capacity. p53 is a potent marker of cancer, with a mutation rate that exceeds 50% in human tumors [21]. Variations in acetylation of p53 could indicate a differential in modulation of protein stability [8] and of transcriptional activity [22]. Acetylation of K164 seems to be important for p53-associated cell growth arrest, and apoptosis, while deacetylation completely abolishes these effects [23]. SIRT1 readily deacetylates p53 and acts as a tumor suppressor, because ablation of SIRT1 results in increased genomic instability in mice [24]. Therefore, low levels of acetylation of p53, as observed in high running capacity rats, could suggest enhanced genetic stability. SIRT1 levels measured the highest in HCR-C rats, which could also help explain the lower levels of acetylated p53 in this group. However, SIRT1 levels decreased in the HCR-T group, which suggests that the activity of the enzyme could be increased by exercise training as has been shown in skeletal muscle of HCR rats [25], but not in LCR rats [16].

p53 protein can regulate apoptosis through Bcl-2 proteins. The Bcl-2 family has pro-and anti-apoptotic roles, BcL-2 itself is a pro-survival protein, while Bax is involved in permeabilization of the mitochondrial outer membrane, causing release of cytochrome *c* and other apoptotic factors into the cytosol [26]. Nonetheless, the changes in Bax and Bcl-2 levels were not significant.

PGC-1α can also be deacetylated by SIRT1 [27]. However,currently there are conflicting data on the involvement of SIRT1 in the activation of PGC-1α. Some reports, [28] including a recent paper from Holloszy’s group suggest that deacetylation of PGC-1α is inhibiting this transcription factor [29], while others indicate that SIRT1-mediated deacetylation activates PGC-1α [30], [31], [32]. It was not necessarily the purpose of the present study to evaluate the interaction between SIRT1 and PGC-1α. However, the changes in protein levels of SIRT1 and PGC-1α were apparently independent.

mtTFA and NRF1 are important transcription factors for mitochondrial biogenesis downstream of PGC-1α. The present data do not indicate that exercise training increases the contents of these transcription factors. In fact, the levels of mtTFA decreased with exercise training in the HCR-T group.

Odf-1 are located in the sperm tail and provide an elastic recoil of the tail, which appears to be important for mobility and protection against shear stress [33]. The levels of spermatogenesis, measured by the content of Odf-1 protein [20], [34], were assessed and exercise training increased the levels of Odf-1 in LCR rats, but to decreasethem in HCR rats. This finding suggests that, in our model, exercise was beneficial for low running capacity animals and increased spermatogenesis, while for high running capacity animals the training impaired spermatogenesis. This finding mimics the bell-shaped dose-response of hormesis [35], suggesting dose dependent effects, although this hypothesis needs further verification. Nonetheless, the changes in LDHC levels support the suggestion that LDHC is a testis-specific isozyme discovered in male germ cells critical for fertilization. This appears to be localized in and on the principal piece of the sperm tail [36]. It has been reported that high levels of chronic exercise training canjeopardize reproductive function in humans [36], [37]. A similar observation was made after 60 weeks of exercise training using a different intensity on 286 subjects [38], suggesting that intensive long term exercise training could have a deleterious effect on reproduction.

## Conclusions

These data suggest that rats with high intrinsic running capacity, through the deacetylation of p53, could have enhanced genetic stability in the testis, which could be beneficial for reproduction. It is suggested that exercise could have a bell-shaped dose-response to exercise in terms of spermatogenesis.
